# Characterization of Cobalt-Based Stellite 6 Alloy Coating Fabricated by Laser-Engineered Net Shaping (LENS)

**DOI:** 10.3390/ma14237442

**Published:** 2021-12-04

**Authors:** Tomasz Durejko, Magdalena Łazińska

**Affiliations:** Institute of Material Engineering, Faculty of Advanced Technologies and Chemistry, Military University of Technology, Sylwestra Kaliskiego 2, 00-908 Warsaw, Poland; magdalena.lazinska@wat.edu.pl

**Keywords:** laser-engineered net shaping, Stellite 6 cobalt-based superalloy, microstructure, microhardness, bending test

## Abstract

The results of microstructure and mechanical properties evaluation of a Stellite 6 (Co-6) alloy deposited on X22CrMoV12-1 substrate by the laser-engineered net shaping (LENS^TM^) technology are presented in this paper. The Stellite 6 alloy is widely used in industry due to its excellent wear resistance at elevated temperatures and corrosive environments. Specific properties of this alloy are useful in many applications, e.g., as protective coatings in steam turbine components. In this area, the main problems are related to the fabrication of coatings on complex-shaped parts, the low metallurgical quality of obtained coatings, and its insufficient adhesion to a substrate. The results of recently performed investigations proved that the LENS technology is one of the most effective manufacturing techniques of the Co-6 alloy coatings (especially deposited on complex-shaped turbine parts). The microstructural and phase analyses of obtained Stellite 6 coatings were carried out by light microscopy techniques and X-ray diffraction analysis. A chemical homogeneity of Co-6 based layers and a fluctuation of chemical composition in coating–substrate zone after the laser deposition were analyzed using an energy dispersive X-ray spectrometer coupled with scanning electron microscopy. The room temperature strength and ductility of the LENS processed layers were determined in static bending tests.

## 1. Introduction

Stellite 6 (Co-6) is one of the most popular cobalt-based alloys exhibiting a good high-temperature oxidation resistance (at temperature up to 1095 °C), excellent thermal stability, good resistance to thermal fatigue, and good resistance to cavitation, corrosion, erosion, abrasion [1,2,3]. The main alloying component of Stellite 6 is chromium (29% in weight), which provides better corrosion resistance and strength by the formation of M7C3, M23C6 carbides [4]. The other components of the alloy are 4.5% W, 1.5% Mo, 1.2% C, Co balance, in weight. Tungsten and molybdenum in Co-6 provide high strength by precipitation hardening. The CoCr-based alloys are used as coating or overlay material in pump seals, bearings, knives seats, blades in aviation engines, nozzle in diesel engines, etc. [5]. 

The hard coating of Stellite 6 is often deposited by conventional welding processes [6] or also the use of surfacing techniques, such as a plasma spray [7,8], high-velocity oxygen fuel (HVOF) [9], and a laser cladding (LC) [10]. Conventional methods (such as surface welding or plasma spraying) are characterized by a high heat input, which results in material embrittlement, distortion, and dimensional instability. Moreover, they are time consuming, difficult to automate, and require relatively large amounts of filling material. Furthermore, the accuracy of these methods is rather low. Gholipour et al. [11] reported that delamination is the dominant mechanism of the wear in Co-6 weld cladding used as coatings, which leads to the necessity of using an intermediate layer between the substrate and a topcoat. However, the coatings obtained by the HVOF method contain oxides and porosity, which can lead to the debonding or spallation of coatings [12]. Therefore, the coatings require additional treatments to improve their properties [13]. The research on Stellite coatings has shown that laser deposition techniques are more effective methods of producing dense and crack-free Stellite 6 coatings [14,15].

Additionally, the increasing performance requirements concerning industrial equipment, especially with regard to surface properties, lead to a situation in which technologies allowing deposition of protective coating and repair of damage elements are granted greater attention. The most effective techniques for producing Co-6 coatings and repairing parts are laser cladding methods [10,16,17,18,19]. The advantages of laser cladding techniques include minimal mixing (a dilution) between a deposit and a substrate, lower heat input and a narrower heat-affected zone, excellent metallurgical bonding between a substrate and clad coatings [20,21]. One of the promising laser techniques for producing protective Co-6 coatings is the laser-engineered net shaping (LENS^TM^) method [22,23]. 

The LENS process that was proposed by Sandia National Laboratories (Albuquerque, NM, USA) and commercially developed by Optomec (Albuquerque, NM, USA) is a novel and innovative additive technology of regeneration/deposition of coatings using a laser beam. As a laser deposition technique of coatings, the LENS technology has several advantages distinguishing it from conventional methods such as welding or thermal spraying. This technology through a layer-by-layer (with controlled thickness) reproduction of a CAD-designed project on the selected area of a metal substrate. Furthermore, the specified type of microstructural morphology (e.g., a columnar structure, a fine-grained, or mixed one), depending on an expected application of alloys can be obtained by using advanced steering and control of heat transfer in the LENS technique [24,25]. In this method, local heating minimizes the width of the heat-affected zone (HAZ), thus reducing the risk of “thermal distortion” and limiting the participation of the substrate material in the deposited layer to below 2% [26,27]. The LENS technique allows for the manufacturing of high-quality thin deposits having little dilution that are nearly 100% dense and with minimal build-up, which lowers the final process costs [23]. 

The LENS technique has so far been used successfully for a compressor seal made of Inconel 718, a reparation of Ti-6Al-4V-bearing housing from a gas turbine engine, depositing the coating with a tungsten carbide alloy on an oil field adapter, and a rotary atomizer used in flue gas desulfurization systems. Previous studies have shown that the LENS technique allows shortening the production/repair time and thus reduces costs by 50%. Moreover, this technique has been used to fabricate a broad range of metallic materials, including stainless steels [28,29], tool steels [30], cobalt alloys [26,31], nickel-based superalloys [28,32,33], copper alloys [34], titanium alloys [35], intermetallic alloys [26,36,37], gradient materials [25,38], and functional composites with improved mechanical material properties, compared with their counterparts obtained by traditional methods. The authors of [39] demonstrated that it is possible to deposit functionally graded alumina coatings on 316 stainless steel substrate using laser-engineered net shaping. Liu et al. [32] used the LENS process for the repair of Ni-base superalloy turbine components that contain casting or manufacturing defects, while Li [40] showed that the LENS system allows a reparation of GTD-111 directionally solidified superalloy. Some other examples include a coating of titanium with tricalcium phosphate (TCP) ceramics to improve bone cell–material interaction [41]. The authors of [42] used the laser deposition of Stellite alloy consisting of 70% Stellite 3 and 30% Stellite 21 to control valve seat sealing surfaces, aiming at enhancing the hardness and wear resistance. The LENS technique has been successfully used for the application of multi-layer Stellite™ coating on stainless steel for use on cutting tools [43]. However, as the authors of [44] have indicated, there are very few papers on the fabrication of Stellite 6 alloy parts using additive techniques. Recently, Traxel and Bandyopadhyay [22] have successfully obtained WC-Co + diamond composites using the LENS technique. Moreover, the prototype sample parts from Co-6 alloy have also been successfully obtained from gas-atomized powder using binder-jet 3D printing [45].

This paper presents the results of technological trials on the deposition of a cobalt-based Co-6 alloy on X22CrMoV12-1 steel substrate (pieces cut off from a new gas turbine blade) using the LENS technology. The coatings obtained were analyzed in terms of obtained microstructures and mechanical properties.

## 2. Materials and Methods

### 2.1. Materials

**Figure 1:**
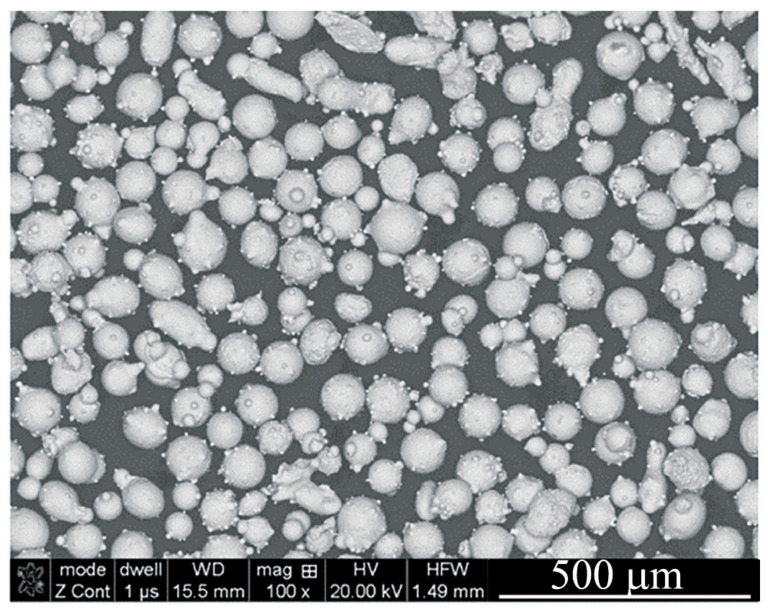
Morphology of Co-6 feedstock powder.

In order to evaluate the possibility of using the LENS technology for the repair of gas turbine blades, Stellite 6 powder purchased from LPW Technology LTD (Gloucester, UK) was used as a batch material. The feedstock material was characterized by a spherical shape and a wide size range of particles (45–150 μm) (Figure 1). Before the deposition process, the powder was dried at a temperature of 200 °C for 24 h under a protective inert atmosphere (a content of oxygen and water vapor was below 0.1 ppm). The brand new turbine (non-exploited) blade made of X22CrMoV12-1 steel was used as the substrate during the laser deposition process. The Co-6 powder was deposited by using the LENS technique on a substrate with dimensions of 15 mm × 150 mm × 10 mm, which was cut by a wire electrical discharge machining (WEDM, ZAP B.P., Końskie, Poland) from the new turbine blade. Then, the substrate was degreased in an ultrasonic bath, dried under a low vacuum for 24 h, and finally blasted.

### 2.2. The LENS Process

The LENS 850-R software (Optomec, Albuquerque, NM, USA) allows the use of a few types of repair processes/repair utilities such as line build deposition, tube/chuck clad deposition, Z clear deposition, and teach-and-learn method. By choosing the adequate method, it is possible to adjust the rebuild method by taking into account the shape of parts. One of them is the teach-and-learn technique. It is used to generate a tool path (series of commands) for depositing material for a part build or repair. The basic idea is to position the deposition head over an area where the material is to be deposited, teach that point, move the deposition head to another location, teach that point, and continue until an area has been taught. The system will then automatically generate the commands needed to deposit material for the lines (contour) defined by the teach-and-learn screen. The area defined can also be filled in (hatched).

The coatings were fabricated by using the LENS 850-R system with different laser power (W) and powder flow rate (RPM) at the constant value of the laser head feed rate (mm/s) (Table 1). The argon flow rates on the central purge and powder nozzles were 25 LPM and 3 LPM, respectively. Tool paths were generated using one of the few variants of the teach-and-learn module, the hatch-fill option. It allows filling the area including its contour (defining all of the characteristic points of the contour is required) by repair material (Stellite 6). During the process, the atmosphere in the working chamber was continuously monitored so that the content of oxygen and water vapor was maintained below 7 ppm. Moreover, in order to avoid crack formation in the applied coatings, before the deposition process, the substrate was heated up to a temperature of 300 °C. This has been confirmed in the work of Liu et al. [32], wherein they noted that cracking is the result of high thermal stresses generated from the LENS processing, and their reduction is possible precisely by preheating the substrate. The research was conducted as part of Grant No. PBS3/B5/37/2015. The aim of this article was to obtain a Co-6 coating without cracks and porosity with a thickness of 0.4 mm at a single deposition pass. The assumed thickness allows reducing the necessity of machining processes.

**Figure 2:**
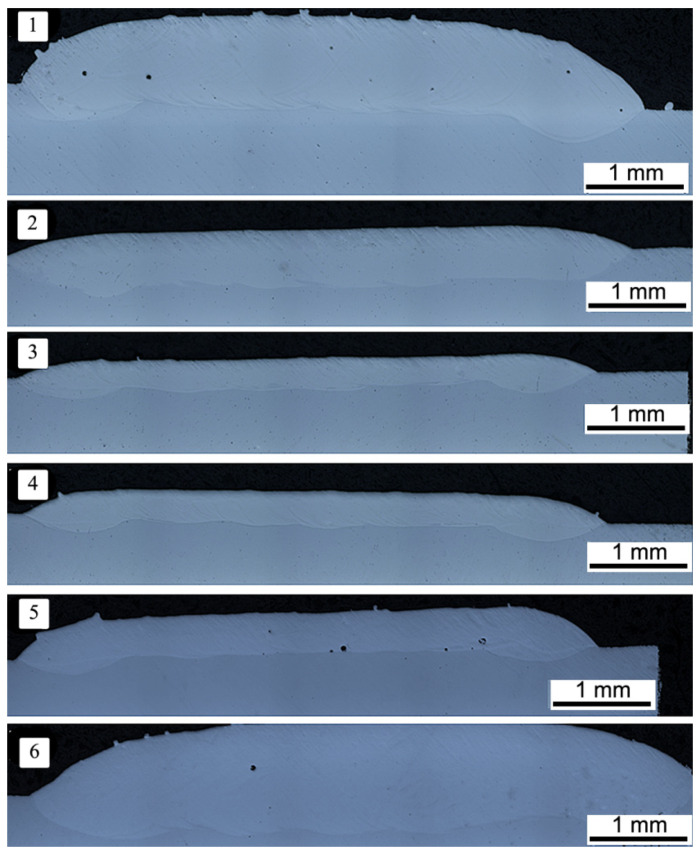
The cross section of Co-6 coatings deposited with different LENS process parameters.

After the preparation of batch material, the set of various technological variants was tested. Table 1 presents the best variants of the performed trials. Based on the visual inspection and performed macroscopic observations, it was found that the Co-6 coatings are characterized by a good metallurgical quality and good adhesion to the substrate material with a clear coating/substrate interface (Figure 2). We have previously shown that the cracks are a common defect that occurs during laser deposition of Stellite 6 superalloy, and it is difficult to avoid [19,46]. During the process of laser deposition of Co-6 alloy, high stresses induced by the high-temperature gradient are generated. Additionally, the cracks can be also produced by some brittle phases/precipitates. Moradi et al. [47] have suggested that process control is required to avoid cracks, for example, by controlling the laser power density.

It should be noted that in the case of variants #2, #3, and #4 (Table 1), there were no discontinuities of structure in a form of pores. For further and detailed analysis, specimen #4 (Table 1) was selected (by taking into account its technological usefulness, including minimal machining allowance). Moreover, it was found that control of a supplied powder amount allows a one-pass deposition with various thicknesses of a deposited layer—starting from that assumed at design step (0.25 mm) up to the value of 1.1 mm (Table 1). It was also observed that the higher laser power at the same powder feed rate increases the thickness of obtained coatings (Table 1). It has been previously documented that [47] increasing the laser power causes more energy to be supplied to feedstock powder, which leads to the melting of its greater volume on the substrate.

Finally, the parameters of coating no. 4 were shown to be suitable. This variant was selected due to low porosity and the thickness fully comparable with the assumed one.

### 2.3. The Characterization Techniques of Coatings

The LENS fabricated samples were cut off by a BP-97d electro-discharge machining device (ZAP B.P., Końskie, Poland) in a perpendicular direction to the substrate. Subsequently, samples were subjected to a metallographic preparation process including grinding with 300–2400 SiC papers and polishing with 3–0.25 µm diamond suspensions. Microstructural details were revealed by a chemical etching with Kallinge’s reagent (50 mL CH_3_OH, 50 mL HCl, 5 g CuCl). 

Microstructure investigations were carried out by Nikon Eclipse MA2000 light microscope (Nikon, Leuven, Belgium) and FEI Quanta 3D field emission gun scanning electron microscope (FEG-SEM, FEI, Hillsboro, OR, USA) equipped with an electron backscatter diffraction (EBSD) system (TSL, Draper, UT, USA) and an energy dispersive spectroscopy (EDS) chemical composition analyzer (EDS, FEI, Hillsboro, OR, USA). Qualitative and quantitative analyses of chemical composition in selected areas of samples were carried out by means of an energy dispersive spectroscopy (EDS) device coupled with an FEI Quanta 3D field emission gun scanning electron microscope. The EBSD technique was applied for some detailed microstructure examinations including size and shape of grains, a phase composition, grain boundaries character distribution, and grains orientation analyses.

The X-ray diffraction (XRD) phase analysis was performed with a Rigaku ULTIMA IV diffractometer (Rigaku, Tokyo, Japan) equipped with a cobalt target (i.e., monochromatic radiation with a wavelength of 0.17889 nm was used). During the diffraction test, CoKα radiation with a voltage of 40 kV and an amperage of 40 mA was used. The analysis was conducted within the 2θ range of 20–160° and with a step size of 0.02°. Obtained results were interpreted by using the DHN PDF 4 crystallographic database (ICDD, Newton Square, PA, USA).

### 2.4. The Mechanical Properties Tests

To validate the effect of a microstructure formed upon the LENS processing on mechanical properties of coatings, the Vickers microhardness distribution measurements were conducted in a perpendicular direction to layers with 50 g load and 10 s loading time in every single indentation. Vickers indentations along five parallel lines (the spacing between lines was 0.5 mm, while the distance of indentations in each line was 0.05 mm) were used in order to carry out microhardness distribution analysis. Based on obtained results, the average values were calculated.

In order to determine the bending strength of the LENS fabricated Co-based alloy coatings, an additional sample was produced by using the LENS process (five samples) according to parameters adopted for variant #4 (Table 1). The Co-6 powder was applied on the previously prepared substrate made of the X22CrMoV12-1 steel with dimensions of 65 mm × 7 mm × 3.5 mm (length × width × height).

The static bending tests were performed by using INSTRON 8501 testing machine (Instron, High Wycombe, UK) at a deformation rate of 2 × 10^−3^ s^−1^. Based on the obtained test, the following parameters were determined:

- A deflection at the maximum bending strength;
- A maximum bending force;
- Bending strength;
- Deformation.

## 3. Results and Discussion

### 3.1. Microstructure of Co-6 Coatings

**Figure 3:**
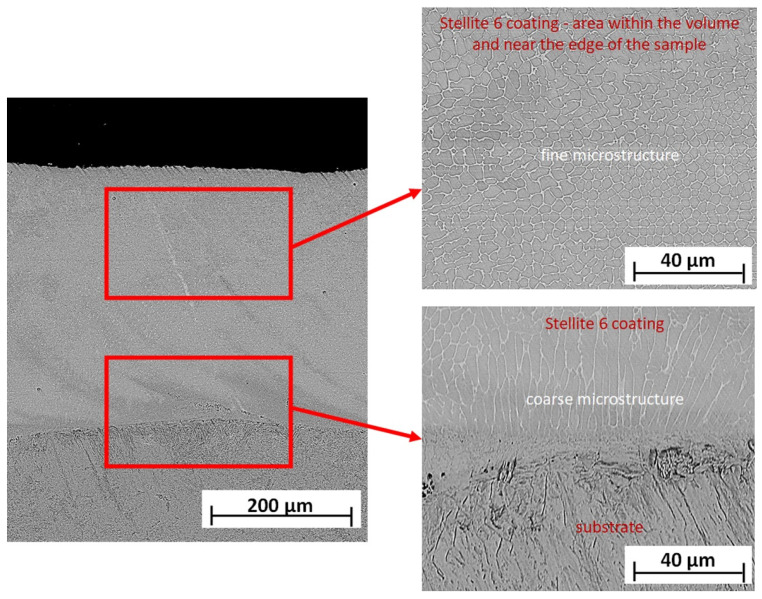
The SEM–BSE images of microstructure of Co-6 coating #4 (according to Table 1).

The microscopic analysis of etched metallographic cross sections of the Co-6 coatings revealed that a microstructure both in a surface layer and entire volume of obtained samples consisted of equiaxed, fine dendrites (solid solution of cobalt) with different sizes (dependent on the distance from the coating surface) and eutectic carbides in the interdendritic spaces (Figure 3). The grain size was analyzed in three characteristic regions, defined as near the edge of the sample, in the middle part of the sample, and near the substrate. According to the results of the SEM analyses, the grain size decreased from the substrate to the edge of the sample. In the area near the substrate, it was the most diverse and equal to 5.5 ± 1.0 μm. It was also observed that the grains near the substrate had an elongated shape. On the other hand, in the sample volume and at the edge, the grain size was 3.7 ± 0.5 μm. The differentiation in grain size in selected three regions of samples resulted from directional heat dissipation in the LENS process, which was presented by Balla et al. [48] and Łazińska et al. [49]. The LENS manufacturing process is assisted by a high-temperature gradient and high cooling rates, which affect the morphology of the microstructure. When applying the first layers of material, material cooling occurs primarily through the cold substrate, which favors the directional growth of grains in the opposite direction of heat dissipation. When the heat transfer through the substrate is no longer dominant, the grains assume a shape similar to equiaxial. The authors of [50] also observed a change in the grain size depending on the analyzed area of the sample, and the grain size decreased from the beginning to the end of samples. In the present case, the grain size decreased from the beginning to the end of samples and was, respectively, about 3 μm at the beginning of the sample and 2 μm at the end. This effect is also explained by the fact that the substrate acted as a heat source.

Moreover, at the distance of about 0.15 mm from the coating–substrate interface, the morphologically changed areas with a banded structure, differently oriented to the substrate, were observed.

**Figure 4:**
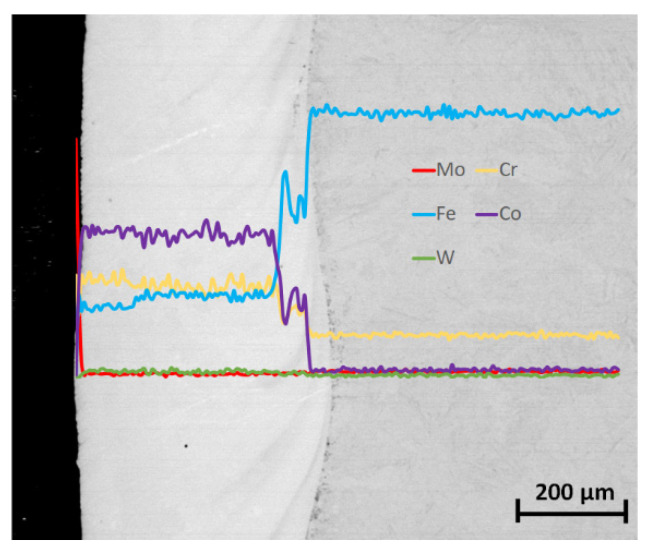
The results of the EDS linear chemical composition analysis of Co-6 coating specimen #4 (according to Table 1).

To determine a fluctuation of chemical composition at the coating–substrate interface, EDS linear measurements were conducted. It was found that the samples produced by the LENS process had a “one-step” transition zone (Figure 4) with an intense increase in elements fraction characteristic for a given alloy.

**Figure 5:**
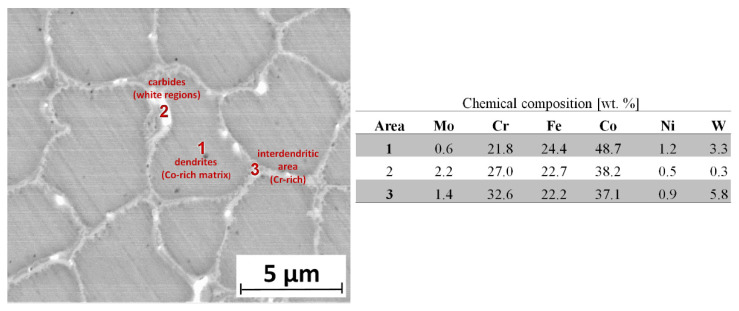
The SEM image shows a microstructure of Co-6 coating #4 (according to Table 1) with the chemical composition of marked areas.

It was also observed, e.g., for variant #4 of the LENS process (Figure 4), that the intense fluctuations of chemical composition occurred, as a result of the “transfer” of a substrate material during the LENS process. It was found that in the analyzed technological variant, the width of the mixed interface from 40 to 100 µm depended on the analyzed area. The cobalt and chromium fluctuations conformed to the passage of the analyzing electron beam through the dispersive CoCr phase, which was also confirmed by the conducted XRD analyses. Moreover, the detailed point analysis of chemical composition performed on the same LENS specimen revealed a presence of the areas with different chemical compositions in the volume of the deposit (Figure 5).

The matrixes of Co-6 deposits fabricated by the LENS method constituted strongly fragmented dendrites (with an average size of 5 µm) based on cobalt (matrix, point #1 in Figure 5), which were separated by interdendritic regions enriched with chromium (grain boundary, point #3 in Figure 5). Interestingly, a third area containing elements such as Cr, Co, and W compositions was also observed. According to the literature, this area corresponds to W-rich carbides. This is the typical hypoeutectic microstructure of the laser-deposited Stellite 6 containing cobalt-rich dendrites and chromium-rich interdendritic areas [10,43]. This type of fine-grained dendritic structure is related to the high cooling rates produced during laser cladding. The same type of microstructure was observed by Thawari et al. [14]. According to them, laser cladding consists of primary dendrites of a Co-rich solid so9lution with Cr-rich interdendritic arrays of M_7_C_3_- and M_23_C_6_-type carbides and the area of W-rich carbides.

### 3.2. EBSD and XRD Phase Analysis

**Figure 6:**
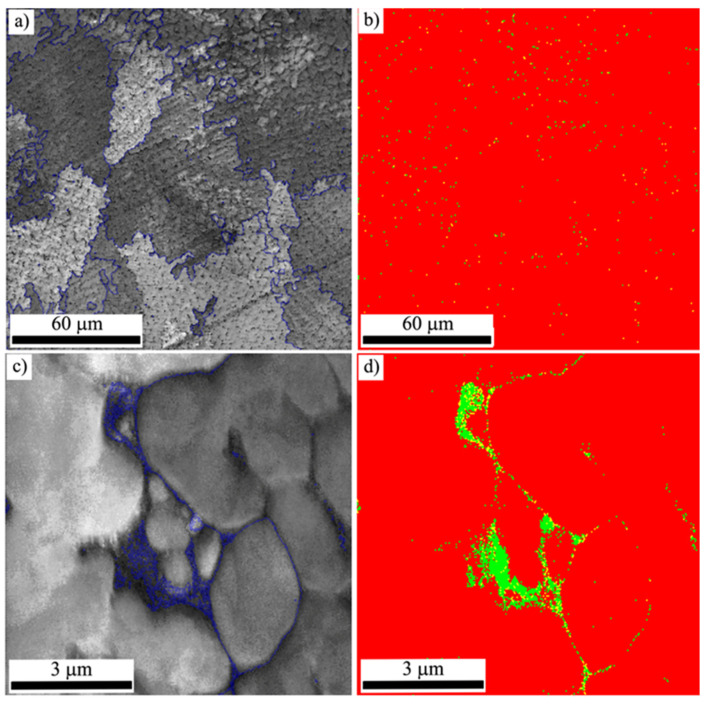
EBSD band contrast map (**a**,**c**) and maps of a phase distribution for sample #4. In (**a**,**c**), the high-angle boundaries (>15°) (grains boundaries’ rotation angle) are marked by blue color. In (**b**,**d**), red color indicates the cobalt austenite phase, and green color indicates the CrCo solution with cobalt–tungsten carbides.

The probable phase composition was estimated based on the analysis of the chemical composition of the coating obtained for variant #4 (Table 1), and it was confirmed by a microanalysis performed by using the EBSD technique (Figure 6). As a result of the obtained phase distribution maps (for two magnifications), the information of the structural components occurring in the Co-6 coating fabricated by LENS was received. It was found that the matrix of the analyzed coating was cobalt austenite, which was decorated by CrCo solution and finely dispersed cobalt–tungsten carbides. Furthermore, a negligible amount of the cobalt–tungsten carbides was detected.

**Figure 7:**
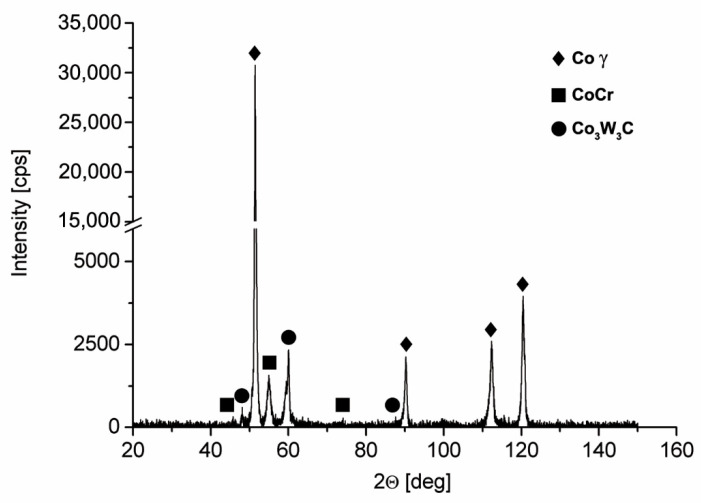
The XRD patterns of the Co-6 coating #4 obtained using the LENS process.

The results of an X-ray microanalysis were confirmed on a macro scale (Figure 7) by using the Rigaku ULTIMA IV diffractometer. It was found that on a macro scale, the phase composition of Co-6 is in good agreement with the results of the electron diffraction in micro areas (EBSD results). The peaks corresponding to the Co phase, the CrCo solution, and the complex cobalt–tungsten carbides (M3C type) were recorded (Figure 7), which confirmed the multiphase structure of the deposit created under applied LENS conditions. The obtained results of the phase analysis correlated with the results of the chemical composition analysis (Figure 5). The X-ray diffraction pattern characterized by high intensity and small width of the identified peaks confirmed also a relatively low level of the internal stresses in the LENS-produced structure deposit.

### 3.3. Microhardness

**Figure 8:**
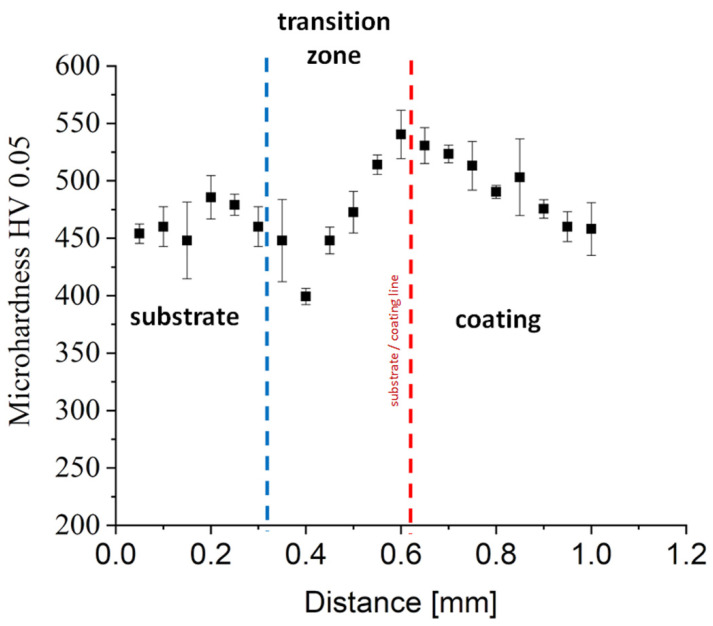
The microhardness distribution of the Co-6 coating #4 deposited using the LENS process.

The obtained microhardness distribution as a function of a distance from the surface for specimen with a coating made of Stellite 6 alloy indicated a partially “tempering” of the subsurface zone of coating at a depth of about 0.4 mm (as an average from the five tracks performed with the same step). This was a result of the lower heat conductivity of protective gas (argon) than a substrate made of X22CrMoV12-1. In this section, a monotonic increase in microhardness was observed until achieving the plateau at a level of about 550 HV (50 HRC) and at a distance of 0.7 mm (Figure 8). At the distance of about 0.7 mm from the coating surface, a decrease in microhardness was observed, reaching 400 HV (41.7 HRC) at a length of approx. 0.2 mm. Probably, substantial microhardness fluctuations occurred due to the presence of hard carbides phases, confirmed by the results of both EBSD and X-ray analyses (Figure 6 and Figure 7). The chart in Figure 8 shows the smooth transition of the microhardness from the laser deposit to the substrate. Traxel and Bandyopadhyay [43] observed a similar effect for Stellite 6 deposited to SS410 substrate using the LENS technique. The Stellite coating obtained by them had the same microhardness, which was 506 HV0.2/15. According to literature data, clad/cast Stellite™ 6 alloys are characterized by hardness in the range ∼450–550 HV [14,42,51]. On the other hand, Thawari et al. [14] have suggested that the large difference in hardness in the zone between the substrate and the deposited layer is related to the increased Fe content [14]. The content of the elements Co and Cr in Stellite 6 at the interface decreases due to the dilution effect. Due to the surface tension and high temperature in the melting zone, the iron diffuses, which results in a lower hardness. Changes in hardness in the heat-affected zone associated with mixing Stellite 6 powders and molted substrate were also observed by the authors of [47].

### 3.4. Three-Point Bending Tests

**Figure 9:**
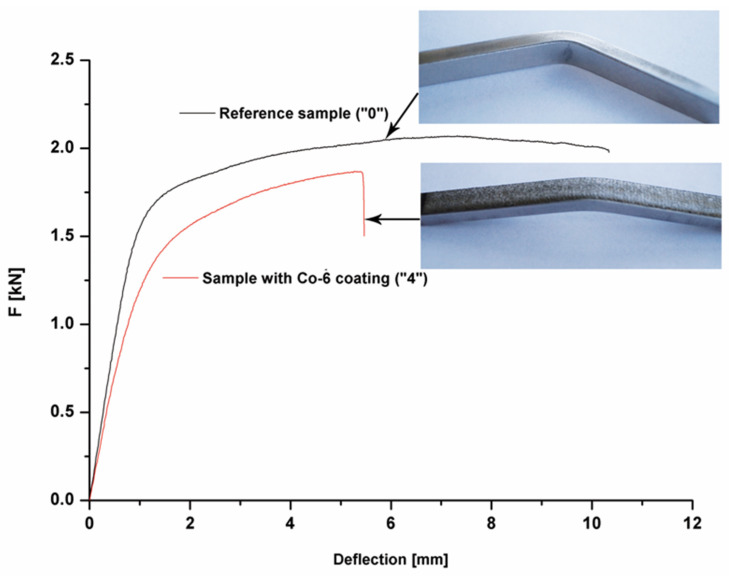
The force vs. deflection curves for the Co-6 coating #4 and without the Co-6 coating #0.

The static bending test was performed for two variants: (I) a sample with a deposited coating made of Co-6 alloy (variant #4) and (II) a reference sample made of a material of turbine blade without coating (the same substrate, marked as #0 in Figure 9). Five samples were prepared for the selected variants. It was found that the substrate material was characterized by a deformation of ~6%. In turn, the bending strength was 1700 MPa. The results of performed bending tests are summarized in Table 2. For the sample with the Co-6 coating produced by using the LENS technique, a decrease in ductility of about 30% was detected, at the statistically comparable bending strength relative to a reference sample (sample “0”). According to the authors of [52], the flexure strength of cobalt-based alloy prepared by the hot isostatic pressing process is 2224 MPa.

**Figure 10:**
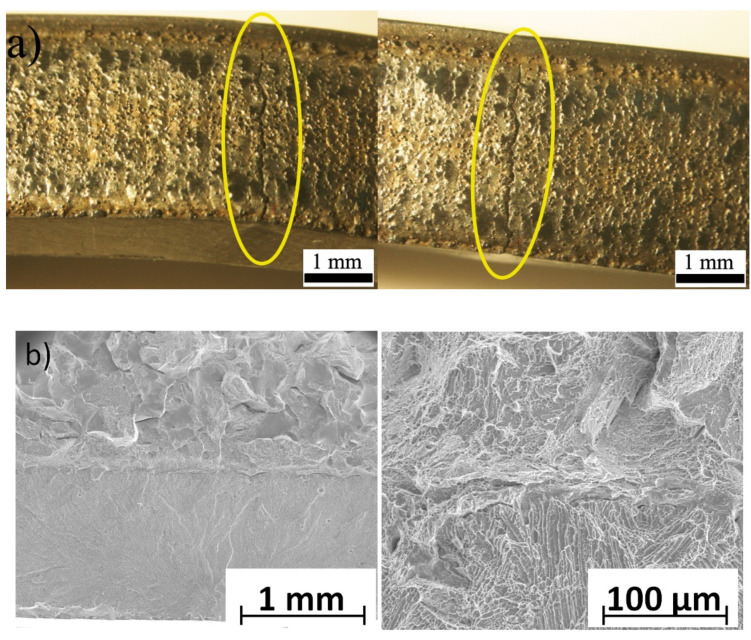
Surface of the Co-6 coating #4 deposited by LENS after the bending test (**a**) and fracture surface of the Co-6 coating, SEM (**b**).

Moreover, the cracks were formed in the coating area without visible interfacial delamination (Figure 10a). The SEM observations revealed no cracks and delamination between the substrate and Co-6 coating (Figure 10b). This proved a durable connection between the substrate material and the deposited coating. Observation of the fractures showed that the type of fracture changed from ductile in the steel substrate to brittle along the interdendritic regions in the Co-6 coating. This effect was also observed by the authors of [53].

## 4. Conclusions

By analyzing the obtained results, it was found that the LENS technique allows deposition of Co-based Stellite 6 coating on the used substrate (fragments of the turbine blade). The proposed alloy after deposition by the LENS technique had a high hardness equal to 50 HRC with a smooth transition zone in the coating–substrate area.

The quality and thickness of the coating deposits were equally influenced by manufacturing LENS parameters—namely, the laser power and powder feed rate.

The best metallurgical quality of the Co-based Stellite 6 coatings was achieved for the parameters of the LENS process ensuring microstructures made of an equiaxed, fine-dendrite-based solid solution of cobalt. The selected manufacturing parameters made it possible to obtain a coating with an assumed thickness of about 0.4 mm when applying one layer of material.

In the case of coatings made of Co-6 alloy marked as #2, #3, and #4 (characterized by different thicknesses), the porosity was not observed. The coatings made of the Co-6 alloy had a characteristic structure for this type of material, which consists of dendrites with an average equal size equal to 5 µm, which are separated by a network of eutectic carbides by chromium-rich interdendritic regions and cobalt–tungsten carbides. The higher dispersion degree of Co-6 coatings was a result of the rapid crystallization of the liquid metal pool due to the interaction of laser radiation.

The obtained Co-6 coating was characterized by the highest hardness near the substrate at the level of 525 HV. At the edge of the coating, the hardness was slightly lower and amounted to 450 HV.

The static bending test showed a decrease in ductility for the sample with the Co-6 coating produced by using the LENS technique, at the statistically comparable bending strength relative to a reference sample. In order to improve the ductility and mechanical properties, an interlayer should be used, which increases the material’s heat capacity during laser beam deposition and reduces the heat dissipation rate, thus leading to an increase in the molten pool. No delamination was observed between the Co-6 coating and the base material after the test.

## Figures and Tables

**Table 1:** The set of the best LENS process parameters used during deposition of Co-6 coatings.

No.	Laser Power [W]	Powder Flow Rate [g/min]	Feed Rate (Contour) [mm/s]	Feed Rate (Hatch) [mm/s]	Thickness of Obtained Coating [mm]	Porosity [%]
#1	600	9.5	10	8	0.94	1.5
#2	600	3.5	10	8	0.55	0.02
#3	400	3.5	10	8	0.25	0.02
#4	400	5.5	10	8	0.40	0.01
#5	400	8.5	10	8	0.44	1.0
#6	800	8.5	10	8	1.10	0.5

**Table 2:** The results of static bending test.

Sample Number	Diamensions of Sample [mm]			Bending Force F [kN]	Deflection [mm]	Bending Strength [MPa]	Deformation [%]
	l_0_	b	H				
#0	50	7	3.5	2.07 ± 0.8	7.13 ± 0.21	1726 ± 69	6.13 ± 0.18
#4	50	7	3.5	1.87 ± 0.37	5.27 ± 1.16	1669 ± 34	4.39 ± 0.09

## Data Availability

The data are available in a publicly accessible repository.
